# Evaluating the Impact of Adopting Green Accounting on Sustainability Reporting at the Central Bank of Iraq (CBI): A Vision to Support the 2030 Sustainable Development Goals (SDGs).

**DOI:** 10.12688/f1000research.175287.1

**Published:** 2026-01-27

**Authors:** Hamzah N. Al-Jumaili, Malik A. Awad, Anwer A. Shahoobe, Ridha A. Abdullah

**Affiliations:** 1 Internal Audit and Control Department, University of Fallujah, Al-Fallujah, Al Anbar Governorate, 31002, Iraq; 2 Internal Audit and Control Department, University of Fallujah, Al-Fallujah, Al Anbar Governorate, 31002, Iraq; 3 Financial Affairs Department, university of Technology, Baghdad, Baghdad, 10066, Iraq; 4 College of Management and Economics, University of Anbar, Ramadi, Al Anbar Governorate, 31001, Iraq

**Keywords:** Green Accounting; Sustainability Reporting; 2030 Vision for Sustainability; Environmental Awareness of senior leaders; the Central Bank of Iraq.

## Abstract

**Purpose:**

This study aims to examine the impact of applying green accounting on the quality of sustainability reports at the Central Bank of Iraq within the framework of the Sustainable Development Goals 2030, It examined the moderating effect of environmental awareness on this relationship.

**Design/methodology/approach:**

A structured questionnaire was used to survey 320 senior officials and accountants regarding green accounting practices, environmental awareness, and reporting quality. Quantitative models using structural equation modeling (SEM) employed to analyze direct and indirect relationships between variables.

**Findings:**

The results showed that green accounting practices had a direct negative impact on the quality of sustainability reports, indicating a discrepancy between objectives and implementation. However, the presence of environmental awareness among leaders reduced the organizational confusion resulting from the new systems represented by green accounting. The results also indicate factors, such as the legal framework and technological maturity, enhancing the effectiveness of green accounting initiatives.

**Practical implications:**

Adopting green accounting without the necessary infrastructure weakens the quality of sustainability reports. A key factor in this is environmental awareness among officials, which promotes transparency and sustainability. Therefore, organizations must understand that the successful implementation of green accounting begins with raising environmental awareness among leaders and employees, training and developing human resources to use these systems, and improving existing technological systems and regulations to align with them and enhance the chances of successful green accounting implementation.

**Originality/value:**

This study makes a pivotal contribution to the academic literature, adding to the limited body of research on the dynamic links between green accounting, sustainability reporting and environmental awareness in banks.

## 1. Introduction

The world faces pressing challenges, most notably climate change, resource scarcity, and environmental degradation. ^9, 17^ These growing challenges have revealed the limitations of traditional financial reporting systems in addressing the environmental impacts of economic activity. In response, green accounting, also known as environmental accounting, has emerged as a strategic framework that integrates environmental considerations into accounting and financial systems as well as planning processes. By identifying environmental costs, such as pollution and resource waste, and directly linking them to responsible activities, green accounting helps highlight and disclose the hidden costs of unsustainable practices. ^17^ This transparency enables organizations and companies to internalize what were previously considered externalities and adopt more sustainable operating strategies. Furthermore, green accounting enables a wide range of stakeholders, including regulators, investors, and the public, to better assess the environmental impacts of economic performance. ^20^ The emergence of green accounting also coincides with a broader shift in corporate and public sector reporting. Traditional accounting has focused primarily on financial metrics, often excluding the environmental and social dimensions of performance. ^17^ However, the growing importance of non-financial performance indicators has led to the development of sustainability reporting, which seeks to measure and disclose environmental, social, and governance factors. In this context, green accounting plays a fundamental role by providing the quantitative environmental data necessary for reliable and standardized sustainability disclosures. ^23^ It aligns with global reporting initiatives such as the Global Reporting Initiative and the Task Force on Climate-related Financial Disclosures, which promote a more holistic view of corporate performance. ^20^ However, despite its growing importance, the field still faces significant obstacles, such as the lack of uniform standards, heterogeneous methodologies, and limited data availability. ^17^ While most available studies have focused on the implementation of green accounting in the private sector and among public government agencies, the role of central banks has not been addressed. However, central banks are in a unique position to lead change in financial policy due to their influence on financial systems, investment flows, and economic policy design. ^12^ Their ability to integrate sustainability principles into monetary and fiscal policy frameworks makes them powerful actors in the pursuit of long-term environmental goals. In the case of Iraq, a country facing significant economic challenges alongside increasing environmental pressures, the Central Bank of Iraq has the strategic potential to act as a catalyst for green transformation. By adopting green accounting, the CBI can enhance its institutional accountability, support national sustainability objectives, and contribute to the achievement of the United Nations Sustainable Development Goals. However, realizing these potential hinges on overcoming a range of challenges, including the absence of standardized reporting systems, limited technical capacity, and weak stakeholder engagement. ^12^

Integrating green accounting principles into accounting and financial systems contributes to improving the quality of sustainability reporting. This is an influential factor in supporting strategic decision making that aligns with the Sustainable Development Goals and Vision 2030. Despite the growing importance of this field, research examining the impact of green accounting on sustainability reporting in central banks remains scarce, particularly in development contexts within the financial sector of central banking institutions. Therefore, this study aims to fill this gap by analyzing the green accounting practices of the Central Bank of Iraq in improving the quality of sustainability reporting, with a focus on assessing their impact on environmental, social, and economic disclosure. It also examines the mediating role of environmental awareness among senior leaders regarding the relationship between green accounting and sustainability reporting. Through this research, we aim to provide theoretical and practical contributions that contribute to enhancing environmental governance practices and financial transparency, thus supporting Vision 2030 for sustainable economic development. The remainder of the study is divided into the following sections: Section Two presents a literature review on green accounting, sustainability reporting, and leadership’s environmental awareness. Section Three describes the methodology. Section Four analyzes the data, exploring the impact of green accounting practices on sustainability reporting, the mediating role of environmental awareness, and examining several control variables. Section Five discusses the results, and finally, a conclusion is presented, including a set of recommendations and prospects for the study.

## 2. Literature review

### 2.1 The concept and evolution of green accounting

Green accounting developed in response to the limitations of traditional accounting in monitoring a company’s environmental costs. As noted by, ^17^ this form of accounting addresses inconsistencies in traditional reporting by assigning monetary value to environmental impacts, thereby promoting more informed financial decision-making. In 2012, Hodi and Hernadi advocated redefining the “going concern” principle in accounting to incorporate environmental sustainability and called for a broader understanding of accounting as a driver of sustainable development. ^11, 20^ also emphasized the importance of standardization in environmental accounting, noting significant differences in environmental reporting across organizations and regions. The results of this study indicate that the lack of effective methods to consistently measure and account for environmental liabilities in financial statements leads to challenges in comparability and data integrity across companies and regions.

### 2.2 Sustainability reporting and the 2030 SDGs

Sustainability reporting is a structured process through which organizations demonstrate their performance in areas such as environmental protection, social justice, and responsible corporate governance. According to a recent study by, ^12^ these reports play an important role in assessing the success of public initiatives, particularly those aligned with the UN 2030 Agenda for Sustainable Development. However, multiple studies have highlighted the discrepancy between declared SDGs and actual implementation, a gap particularly acute in developing countries. As ^1^ note, mandatory reporting often lacks awareness, uses inconsistent benchmarks, and suffers from a lack of institutional support. This challenge is particularly acute in developing countries such as Iraq, where public entities face numerous constraints in aligning their tax systems with the SDGs. Furthermore, ^16^ note that small and medium-sized enterprises face difficulties in reporting due to limited resources and expertise obstacles that can also hinder larger public institutions such as central banks. In this sense, Iraq’s Vision 2030 aims to achieve good governance through anti-corruption and transparency two key factors for an efficient industrial sector, as emphasized by. ^2^ To achieve this, the vision envisages the establishment of mechanisms, such as the creation of a National Sustainable Development Council, to ensure participation and strategic future planning, as outlined in the Multi-stakeholder Report ^15^ Therefore, achieving the 2030 Sustainable Development Goals through effective reporting requires not only political mandate but also institutional capacity building and stakeholder engagement.

### 2.3 The role of Central banks in promoting sustainability

Central banks play a paramount role in shaping economic behavior and promoting sustainability. As ^23^ emphasize, financial institutions can lead the system toward more environmentally conscious practices by integrating sustainability into their frameworks ^12^ find that public environmental accountability improves when accounting is systematically approved. Their research suggests that, despite some policy progress, a lack of transparent non-financial reporting limits accountability. This finding resonates with central banks, which, regardless of their commercial status, manage significant financial flows consistent with Vision 2030 goals.

Economic reconstruction and institutional solidification remain top priorities in Iraq, and central banks can provide fiscal stimulus to drive the green transition ^17^ note that this requires clear benchmarks, stakeholder collaboration, and a synchronized, long-term focus to ensure substantial results ^3^ note that central banks have begun exploring how to use monetary policy tools to promote the transition, for example, by purchasing green assets through asset purchase programs, as some central banks did in 2022. There are also incentives such as low-interest financing for renewable energy projects, in line with a 2020 move by the Central Bank of Iraq. ^4^

## 3. Methodology

### 3.1 Informed consent

The participants were adults of sound mind and legal capacity, and they provided their informed verbal consent before participating. Because the participants were senior and middle-level officials and accountants at a sensitive financial institution (the Central Bank of Iraq), the verbal consent was instrumental in increasing the response rate, as it reassured participants that their identities would not be linked to their professional answers in any written record. They were informed of the study’s purpose and nature, and that their questionnaire responses did not include any personal information, nor did they relate to any health or medical issues or contain any discriminatory statements. Rather, their responses pertained to a professional topic concerning green accounting and financial reporting at the Central Bank of Iraq.

### 3.2 Ethical considerations

The information contained in this research is complete and accurate and does not violate internationally recognized laws and ethics, as approved by the Research Ethics Committee. This research has received approval from the Research Ethics Committee of the College of Administration and Economics, a constituent college of the University of Fallujah, under number [UOF.HUM.2025.001].

### 3.3 Research model and hypotheses

**Figure 1.**
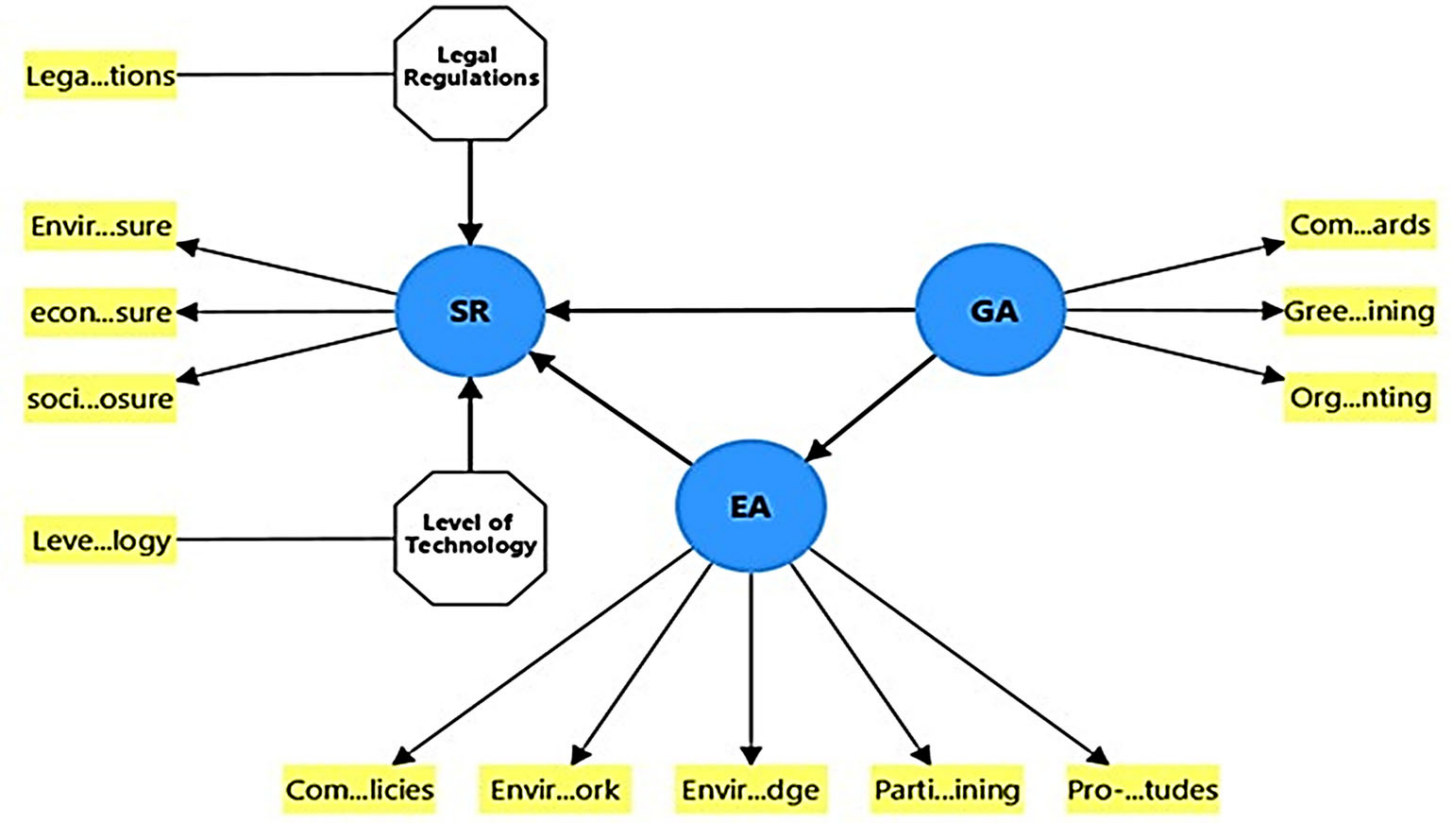
Conceptual model.

This theoretical framework examines the impact of green accounting practices on sustainability reporting. Sustainability reporting encompasses the disclosure of environmental, social, and economic information, as well as compliance with standards such as the Global Reporting Initiative (GRI) and the Sustainable Development Goals (SDGs). Reports also consider corporate transparency, accountability, and the integration of sustainability into annual reports. Figure 1 shows the relationships between the variables in the theoretical model.

Regardless of regional and sector differences, past studies have made remarkable discoveries concerning the effects of green accounting on sustainability reporting. For example, the use of both primary and secondary data, as well as the deployment of multiple research methods, are legacies left behind by scholars from many developing countries, including Indonesia, Bangladesh, and the Republic of Ghana, and Vietnam.

Reference [ 14] as well as, ^21^ are among the most notable authors who conducted quantitative studies and established a strong positive relationship between the implementation of green accounting practices (e.g. the use of recycled materials, environmental cost allocation, and investing in renewable energy) and the quality of sustainability reports. These studies affirm that the use of such practices improves the credibility of environmental information and enhances disclosure and transparency. Furthermore, ^8^ indicated that social responsibility reporting may mediate and strengthen the linkage between environmental accounting and sustainable development which illustrates the interrelationship between the environmental and social aspects of the reporting system. In the Ghanaian pharmaceutical industry, ^22^ discovered that using the PLS-SEM model, eco-efficiency, adherence to environmental legislation, and environmental costs had a positive and significant effect on the sustainability performance of the industry.

Reference [ 19] along with ^5^ have conducted more conceptual and qualitative analyses support the need for fully developed regulatory frameworks (including the GRI and TCFD standards) as well as the necessary integration of high-quality economic and ecological environmental accounting. They also analyze the organizational and cultural elements that influence the effective practice of green accounting.

As indicated by, ^10^ product-design as well as activity-based accounting that utilizes life cycle costing can capture a balance between environmental and economic efficiency and suggests that green accounting needs to be adopted as part of the accounting framework for sustainable business models. Research by ^6, 23^ argue that industry-specific standardized frameworks and practices for SMEs can optimize reporting and boost competitive edge. Overall, these studies agree that green accounting is fundamental for enhancing sustainability reporting, reconciliation of ecological and economic sustainability, provision of institutional frameworks, appropriate regulation, and precise measurement and analytical methods. The guiding hypotheses are outlined below.

H1: Adoption of green accounting Positively affects the quality of sustainability reporting.

Environmental awareness among senior leadership is a key factor in enhancing the effectiveness of environmental accounting practices (in terms of the quality of sustainability reporting). Numerous studies have shown that environmentally conscious leadership whether through executive sponsorship or a diverse board of directors significantly impacts the success of environmental accounting implementation and the effectiveness of reporting.

For example, a detailed study of Chinese manufacturing companies found that executive sponsorship can reduce perfunctory or sloppy environmental management practices and promote genuine environmental accounting implementation. A global study showed that CEO independence alone has a significant positive impact on the quality of sustainability reporting, while the independence of other board members has no significant effect.

An independent study analyzing companies in the French SBF 120 index showed that gender diversity on the board, particularly the presence of female directors, is associated with improved environmental reporting quality. Research in the Asia-Pacific region suggests that the role of the board in sustainability reporting can be limited in certain organizational and cultural contexts, highlighting the importance of environmental factors for effective environmental governance. The most important governance variables associated with reporting quality are senior management support for environmental accounting adoption, an autonomous CEO, gender diversity in corporate governance, and environmental awareness among senior management. As observed in some South African companies, these variables sometimes weaken the positive relationship between integrated reporting and sustainability performance. These findings confirm that the relationship between environmental accounting adoption and reporting quality is not straightforward but rather influenced by leadership characteristics, organizational dynamics, and the environmental context. Therefore, sustainability researchers and practitioners must consider leadership dimensions as a key factor in the success of environmental reporting initiatives and in promoting environmental transparency and accountability. Based on previous research, the guiding hypotheses are as follows:

H2: Senior leaders’ environmental awareness influences the relationship between green accounting adoption and sustainability reporting quality.

### 3.4 Population and sampling procedure

The research sample was the personnel of the Central Bank of Iraq, specifically those in the accounting and environmental management departments. From the purposive sample, 320 individuals with the necessary experience and knowledge were selected to respond to the questionnaire. To enhance data accuracy, senior bank employees were also asked questions regarding the mediating variable, “environmental awareness.” The data collected from these individuals formed the unit of analysis.

**3.4.1 Sample description and demographic characteristics**

The sample characteristics reported in this study include gender, age group, educational level, years of professional experience, job title/grade, and organizational department. Table 1 shows the detailed distribution of the sample’s demographic characteristics.

**Table 1.** Demographic characteristics of the sample.

Variable	Categories	Number	Percentage (%)
Gender	Males	200	62.5%
	Females	120	37.5%
Age Group	Under 30	40	12.5%
	30–39	100	31.3%
	40–49	110	34.4%
	Over 50	70	21.8%
Educational Level	Bachelor’s	160	50.0%
	Master’s	100	31.3%
	PhD	60	18.7%
Years of Experience	Less than 5 years	30	9.4%
	5–10 years	80	25.0%
	Over 10 years	210	65.6%

The table shows that the majority of participants were male (62.5%), and that most of them belonged to the middle and older age groups (30–49 years, 65.7%), in addition to a significant percentage of those over fifty (21.8%). In terms of education, bachelor’s degree holders represented nearly half of the sample (50%), followed by master’s degree holders (31.3%) and doctorate holders (18.7%). As for practical experience, participants were predominantly long standing, with nearly two-thirds of them having more than ten years of experience (65.6%).

### 3.5 Developing the study tool

The research instrument (questionnaire) was developed to measure the primary research variables, in addition to the control variables: green accounting adoption (independent variable), sustainability reporting (dependent variable), and managers’ environmental awareness (mediating variable). To ensure the accuracy of the instrument, it underwent three phases: preliminary drafting (prepared based on previous research and theoretical frameworks); scientific review (submitted to experts for revision); and pilot study (administered on a small sample to ensure item clarity). The questionnaire used a five-point Likert scale (ranging from “strongly disagree” to “strongly agree”). The appendix provides detailed information on the variables, dimensions, and number of items (see Table 2).

**Table 2.** Research variables.

Type	Variable name	Code	No. of items
**Independent**	**Adoption of Green Accounting**	**GA**	**15**
	Compliance with Environmental Accounting Standards	GA_CA	1-5
	Organizational Structure for Green Accounting	GA_OS	6-10
	Green Accounting Training	GA_TR	11-15
**Mediating**	**Environmental Awareness of senior leaders**	**EA**	**25**
	Environmental Knowledge	EA_EK	16-20
	Pro-Environmental Attitudes	EA_PA	21-25
	Environmental Behavior at Work	EA_EB	26-30
	Commitment to Green Policies	EA_CP	31-35
	Participation in Environmental Training	EA_PT	36-40
**Dependent**	**Sustainability Reporting**	**SR**	**15**
	Environmental disclosure	SR-END	41-45
	Social disclosure	SR-SD	46-50
	Economic disclosure	SR-ECD	51-55
**Control**	**Legal Regulations**	**CV_LR**	**56-60**
	**Level of Technology**	**CV_LT**	**61-65**

### 3.6 Data collection procedures

The study used a paper questionnaire that included demographic data and questions on variables such as environmental accounting, managerial environmental awareness, and sustainability reporting. The primary participants were senior officials and staff from the administrative and accounting departments of the Central Bank of Iraq. The questionnaire was distributed directly to the target group, resulting in 320 complete questionnaires. The responses were reviewed and converted into Excel spreadsheets for statistical analysis.

### 3.7 Analytical framework (Quantitative)

The analysis in this study relies entirely on quantitative analysis to test hypotheses and assess relationships between variables. Data is analyzed using Smart-PLS statistical software, which is widely used in structural equation modeling (SEM) using partial least squares. The analysis includes measuring the reliability and validity of the model, as well as testing the causal relationships between the adoption of green accounting and improved sustainability reporting.

### 3.8 Model specifications

This analysis employed structural equation modeling (SEM) and regression analysis. SEM is a powerful mediation analysis method. It can examine the relationship between an independent variable (green accounting practices) and a dependent variable (sustainability reporting) and determine whether one or more mediating variables (managers’ environmental awareness) influence this relationship. This analysis was based on SEM due to its flexibility in mediation analysis. It allows for the inclusion of multiple variables in the model and the simultaneous estimation of direct and indirect effects, while accounting for measurement error (See Figure 1).

The following three models are presented. The first model describes the overall effect of the independent variable on the dependent variable. The second model describes the effect of the independent variable on the mediating variable. The third model measures the direct effect of the combined independent and mediating variables on the dependent variable. These models form the core of Baron and Kenny’s method for measuring mediation effects and help determine whether the direct effect disappears or is significantly weakened after the introduction of the mediating variable, thereby confirming the existence of a mediation effect.

$$\mathrm{SR}=a_{0}+\beta_{1}\mathrm{GA}+\varepsilon$$ (1)

$$\mathrm{EA}=a_{0}+\beta_{2}\mathrm{GA}+\varepsilon$$ (2)

$$\mathrm{SR}=a_{0}+\beta_{1}\mathrm{GA}+\beta_{3}\mathrm{EA}+\varepsilon$$ (3)

## 4. Data analysis

### 4.1 Outer model/Measurement model

4.1.1 Outer loadings

The data in Table 3 demonstrates the strength of the correlations between the manifest indicators and the latent variables in the PLS-SEM model using four key statistics: the original sample (O), the replicated sample mean (M), the standard deviation (STDEV), and the t-statistics. The “Green Political Commitment” indicator appears to be one of the most strongly correlated indicators with the latent variable “Environmental Awareness (EA).” Its loading value is high at 0.938, with a low standard deviation of 0.005, reflecting the homogeneity of the estimates. Its t-value is approximately 191.486, indicating a high degree of statistical significance. The “Compliance with Environmental Accounting Standards” indicator, which is related to “Environmental Accounting (GA),” has a loading value of 0.554, a standard deviation of 0.055, and a t-value of 10.039, also indicating significance.

**Table 3.** Outer loadings.

	Original sample (O)	Sample mean (M)	Standard deviation (STDEV)	T-statistics (|O/STDEV|)
Commitment to Green Policies <- EA	0.938	0.938	0.005	191.486
Compliance with Environmental Accounting Standards <- GA	0.554	0.551	0.055	10.039
Economic disclosure <- SR	0.835	0.835	0.022	38.149
Environmental Behavior at Work <- EA	0.613	0.611	0.044	13.955
Environmental Disclosure <- SR	0.876	0.876	0.011	81.760
Environmental Knowledge <- EA	0.801	0.800	0.018	44.491
Green Accounting Training <- GA	0.877	0.878	0.012	73.656
Organizational Structure for Green Accounting <- GA	0.916	0.915	0.014	65.849
Participation in Environmental Training <- EA	0.840	0.841	0.015	55.286
Pro-Environmental Attitudes <- EA	0.354	0.352	0.060	5.901
Social disclosure <- SR	0.744	0.742	0.031	23.620

Other indicators also achieved high values, highlighting the validity of the model. These indicators include “Economic Disclosure,” related to Sustainability Reporting (SR), with a load of 0.835, “Environmental Reporting,” with a loading of 0.876, and “Green Accounting Training,” with a loading of 0.877. These indicators all exhibited high t-values, reinforcing their significance. Indicators such as “Environmental Knowledge,” “Participation in Environmental Training,” and “Green Accounting Organizational Structure” also exhibited loadings between 0.80 and 0.91, with low standard deviations and high t-values, reflecting the high quality of the latent variable representation. Overall, the consistency of values between (O) and (M), the low standard deviations, and the high t-values demonstrate the reliability of the model and the accuracy of the relationships between the indicators and the variables they represent, thus supporting the theoretical framework used in this study.

**4.1.2 Construct reliability and validity**

Table 4 presents the reliability and construct validity consistency assessment results for a set of latent variables. Three primary assessment tools were used: Cronbach’s alpha, composite reliability, and average variance extracted (AVE). Internal consistency and convergent validity were also qualitatively assessed.

**Table 4.** Construct reliability and validity.

Construct	Cronbach’s alpha	Composite Reliability (CR)	Average Variance Extracted (AVE)	Internal consistency evaluation	Convergent validity evaluation
**EA** (Environmental Awareness)	0.778	0.847	0.545	Good	Acceptable
**GA** (Green Accounting)	0.705	0.835	0.638	Good	Good
**SR** (Sustainability Reporting)	0.754	0.860	0.672	Very Good	Very Good

The reliability of the “Environmental Awareness (EA)” variable was 0.778, within the acceptable range, indicating good internal consistency. Composite reliability (CR) reached 0.847, exceeding the minimum acceptable value of 0.7, indicating that the scale was consistent. The average variance extracted (AVE) was 0.545, within the acceptable range and exceeding the minimum acceptable value of 0.5, supporting convergent validity.

For “Green Accounting (GA),” α = 0.705 and CR = 0.835, both reflect good internal consistencies. AVE = 0.638 reflects the ability of the indicators to explain a sufficient proportion of the latent variable’s variance, making both internal consistency and convergent validity for this dimension “good.”

“Sustainability Disclosure (SR)” showed relatively higher results, with α = 0.754, CR = 0.860, and AVE = 0.672. These values indicate a high degree of reliability and stability, justifying the rating as “very good” in terms of both internal consistency and convergent validity. Thus, the indicators used for each of the three variables support the theoretical framework and enhance the reliability and accuracy of the study’s latent model results.

**4.1.3 Coefficient of determination**

Table 5 shows the coefficient of determination (R ^2^) of the explanatory power of the independent variables in the model on the dependent variable. For the variable “Environmental Awareness” (EA), the raw and adjusted R ^2^ values are 0.913, indicating that 91.3% of the variance in EA is explained by the independent variables. This demonstrates the model’s strong explanatory power, supported by the narrow confidence interval (0.891-0.935). For the variable “Sustainability Disclosure” (SR), the raw R ^2^ value is 0.718, and the adjusted R ^2^ value is 0.717, indicating that the model explains 71.8% of the variance, demonstrating strong explanatory power. These results reflect the model’s ability to explain the overall behavior of the dependent variable.

**Table 5.** Coefficient of determination.

Dependent variable	R ^2^-(Original sample)	95%-Confidence interval	R ^2^ Adjusted	Interpretation
Environmental Awareness (EA)	0.913	0.891 – 0.935	0.913	Excellent explanatory power
Sustainability Reporting (SR)	0.718	0.681 – 0.757	0.717	Strong explanatory power

**4.1.4 Effect size (f** ^**2**^ **)**

Table 6 shows the effect sizes (f ^2^) of the independent variables on the dependent variable in the model. The path from GA to EA has a high effect size of f ^2^ = 10.474, an outlier, indicating that green accounting (GA) has a strong influence on environmental awareness (EA). The high confidence interval (8.165–14.276) supports this conclusion. The path from EA to SR also exhibits a large effect size of f ^2^ = 0.542, indicating that environmental awareness has a strong influence on sustainability reporting (SR). The effect size of GA on SR is f ^2^ = 0.081, which is within the smaller range, indicating a weak effect, but is statistically significant. These results reflect the varying strengths of the various relationships in the model, with the greatest influence concentrated on the middle path. (GA → EA → SR).

**Table 6.** Effect size (f ^2^).

Path	f ^2^ (Original sample)	Confidence interval (2.5% - 97.5%)	Interpretation of effect size
GA → EA	10.474	8.165 – 14.276	Very large effect
EA → SR	0.542	0.425 – 0.681	Large effect
GA → SR	0.081	0.036 – 0.153	Small effect

### 4.2 Direct and indirect effects and hypothesis testing results

**Figure 2.**
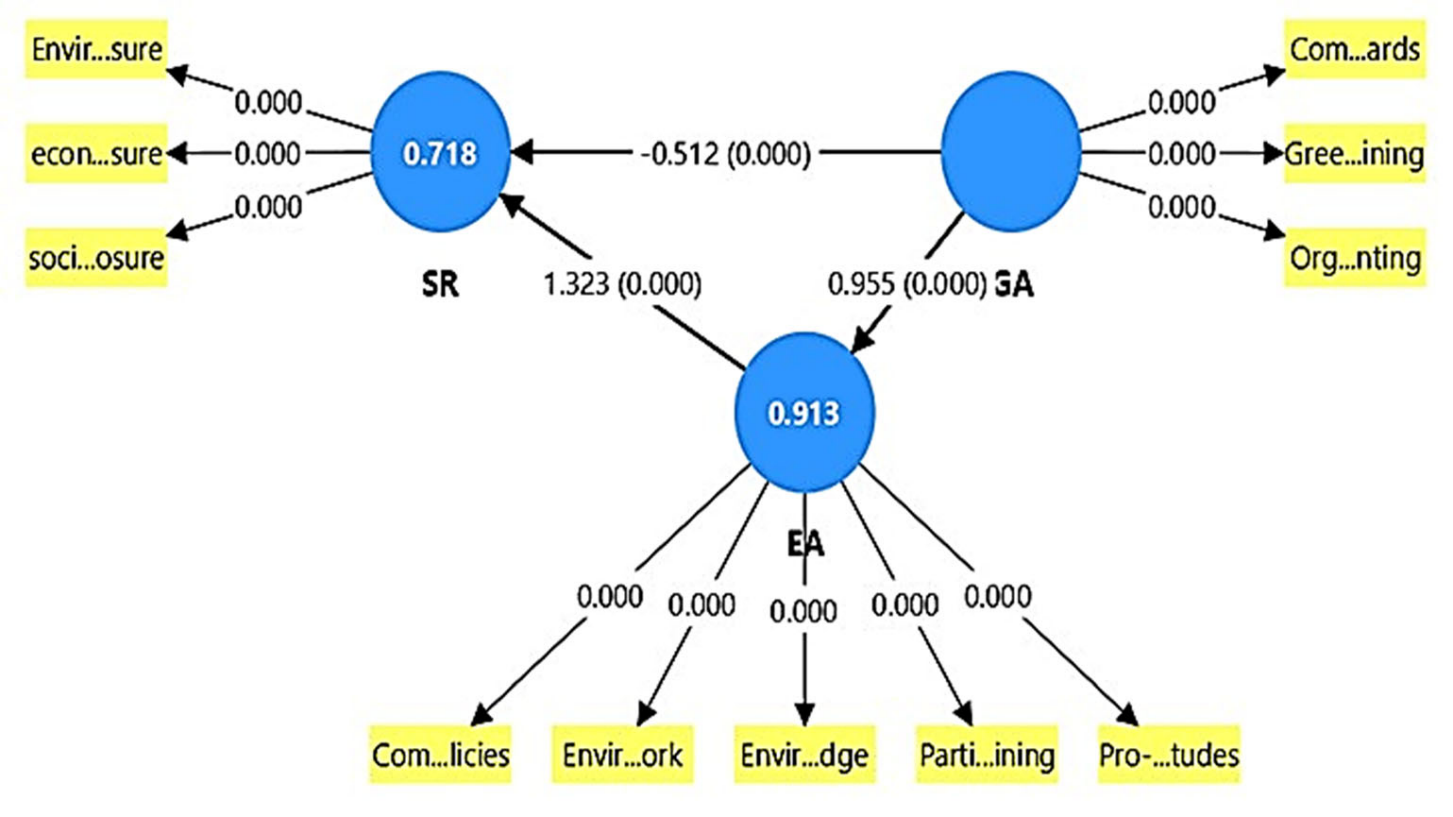
Direct and indirect Effects results.

Based on path analysis, the statistical results in Table 7 and Figure 2 indicate that the adoption of green accounting (GA) has multiple impacts on sustainability reporting quality (SR), both directly and indirectly through managers’ environmental awareness (EA). The results indicate that the adoption of green accounting has a direct, significant, negative impact on SR quality, with an effect coefficient of -0.512 and a t-value of 4.584, statistically significant at the P = 0.000 level. This indicates that the first hypothesis (H1: “The adoption of green accounting has a positive impact on SR quality”) is not consistent with the actual data and is therefore rejected. The results indicate that the opposite, significantly negative, impact is not the hypothesized positive effect. This suggests that directly adopting green accounting without strengthening environmental awareness may lead to a decline in SR quality due to the lack of formality or maturity of these practices. However, the results indicate that the introduction of green accounting has a significant, direct, positive impact on managers’ environmental awareness. The effect coefficient is 0.955, with a t-value of 162.413, statistically significant at the P = 0.000 level. This indicates that these practices have effectively increased managers’ awareness and attention to environmental issues. In turn, this awareness has a strong direct effect on the quality of sustainability reporting (1.323, t = 12.789, p = 0.000), highlighting the importance of environmental assessment as a mediating variable. This finding can be interpreted from a resource theory perspective as meaning that mere adoption is insufficient to demonstrate that implementing green accounting will improve the quality of sustainability reports. Banks must possess robust resources and sophisticated management and organizational leadership to translate new practices specifically, the implementation of green accounting into tangible positive outcomes. ^18^ On the other hand, the negative result aligns with organizational change theory, which posits that the actual impact of environmental practices on organizational change depends on complex dynamics and factors, including the interaction of resources, capabilities, and leadership awareness, rather than solely on the symbolic adoption of green accounting. ^7, 13^ Furthermore, the studied society suffers from a lack of incentives to support sustainability, in addition to limited dynamic capabilities to translate environmental practices into tangible performance. Iraqi banks, and public institutions in general, often adopt such practices symbolically without linking them to their operational strategies or integrating them into their organizational culture. This context leads to negative or ineffective outcomes for the quality of sustainability reports; Therefore, the study identifies the crucial factor as the degree of environmental awareness, the dynamic element through which green accounting practices can successfully produce high-quality, well-organized sustainability reports.

**Table 7.** Direct and indirect effects.

Path	Effect type	Original sample (β)	T-Statistic	P-Value	95% confidence interval	Interpretation
GA → EA	Direct Effect	0.955	162.413	0.000	0.944 – 0.967	Very strong and significant direct effect
EA → SR	Direct Effect	1.323	12.789	0.000	1.149 – 1.554	Very strong and significant direct effect
GA → SR	Direct Effect	-0.512 (previously)	4.584	0.000	(from earlier)	Significant negative direct effect
GA → SR	Indirect Effect	1.264	12.130	0.000	1.090 – 1.496	Significant positive indirect effect (via EA)
GA → SR	Total Effect	0.752	36.305	0.000	0.711 – 0.792	Positive overall effect combining direct & indirect

**Table 8.** Hypothesis testing summary.

Hyp.	Statement	Path tested	Result	Conclusion
H1	Adoption of green accounting positively affects the quality of sustainability reporting.	GA → SR (Direct Effect)	β = -0.512, T = 4.584, p = 0.000 (Significant negative effect)	Rejected
H2	Senior leaders’ environmental awareness influences the relationship between green accounting adoption and sustainability reporting quality.	GA → EA → SR (Indirect Effect)	Indirect effect = 1.264, T = 12.130, p = 0.000 (Significant positive mediation)	Accepted (Mediating role confirmed)

Source: Prepared by the authors.

Analysis of the indirect effect of green accounting adoption on sustainability reporting quality through environmental awareness revealed a significant positive indirect effect of 1.264. Combining the direct and indirect effects yields a highly significant positive effect overall (0.752). This confirms that green accounting adoption only exhibits a positive overall effect when environmental awareness is high.

The results support the second hypothesis (H2: “Managers’ environmental awareness influences the relationship between green accounting adoption and sustainability reporting quality”), as this awareness plays a key role in transforming the impact of green accounting adoption from a negative one (in the absence of environmental accounting) to a positive one (in the presence of environmental accounting). This suggests that environmental awareness effectively mediates this relationship.

### 4.3 Control variable

**Figure 3.**
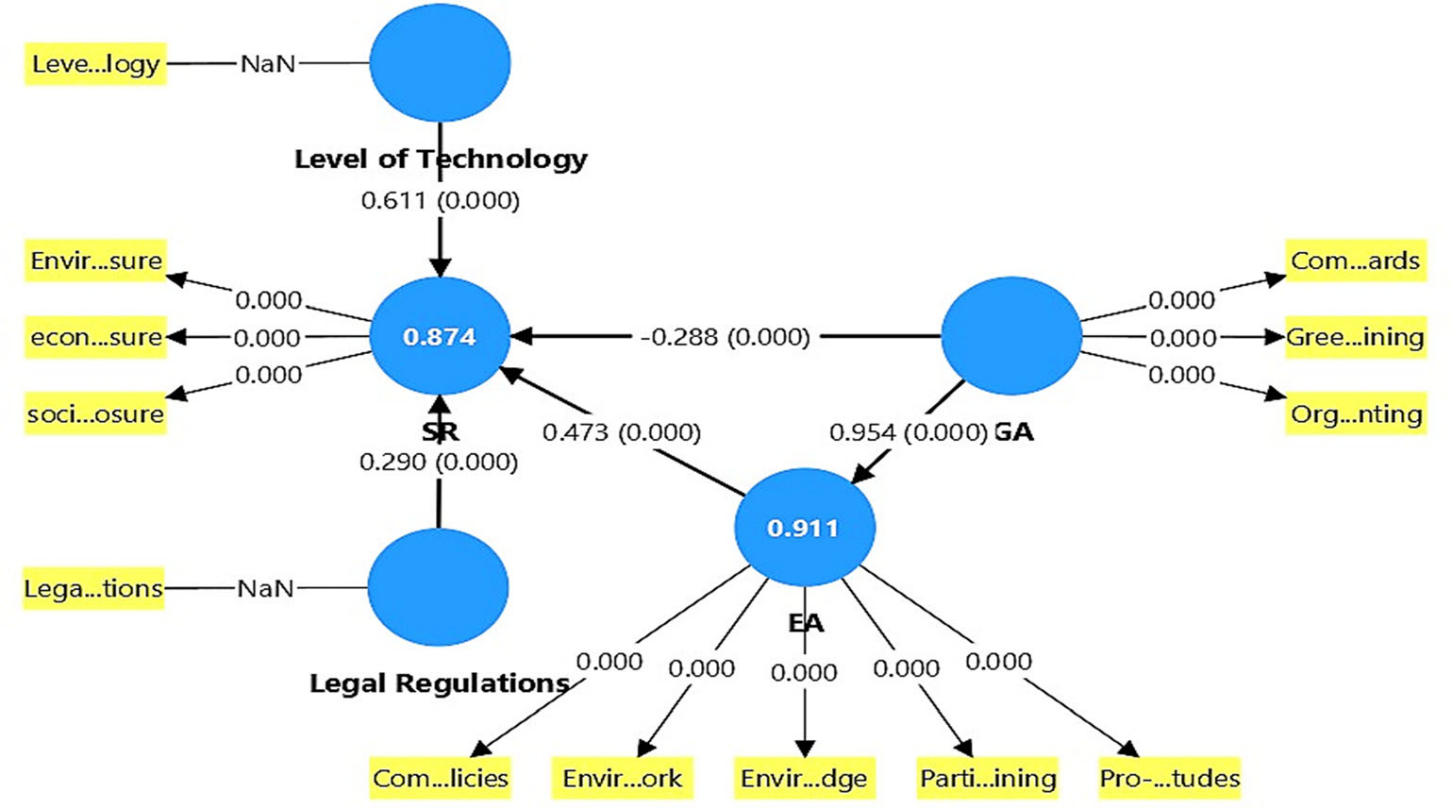
Results of the impact of control variables. Source: Prepared by the authors.

The results in Tables 9- 10 show that the inclusion of the control variables (legislation and technology level) in the model of the relationship between green accounting, environmental awareness, and sustainability reporting quality has significant effects, contributing to a comprehensive understanding of the factors influencing reporting quality. Legislation has a significant direct effect on sustainability reporting quality, with an f ^2^ value of 0.191, which is considered moderate according to Cohen’s criteria. This suggests that the presence of a supportive legal framework helps improve sustainability reporting quality by strengthening institutional commitment to information disclosure and accountability. Technology level has a very strong direct effect on sustainability reporting quality, with an f ^2^ value of 1.223, which is very high, indicating that the use of advanced technologies (such as intelligent accounting systems or digital reporting systems) plays a crucial role in improving the efficiency and quality of sustainability reporting. Regarding the model’s explanatory power (R ^2^), the inclusion of these control variables increases the R ^2^ value of the dependent variable, sustainability reporting quality (SR), from 0.718 to 0.874. This indicates that these variables significantly enhance the model’s explanatory power and help explain variations in reporting quality. In contrast, the explanatory power of environmental awareness (EA) was not significantly affected (decreased from 0.913 to 0.911), meaning that these variables did not directly or significantly influence this variable. See Figure 3.

**Table 9.** Effect size (f ^2^).

Path	f ^2^ (Effect size)	T-Statistic	P-Value
Legal Regulations → SR	0.191	3.800	0.000
Level of Technology → SR	1.223	7.859	0.000

**Table 10.** R-square (power of explanation).

Dependent variable	Before adding controls	After adding controls	Note
EA	0.913	0.911	Did not change much
SR	0.718	0.874	Significant increase in explained variance

## 5. Results and discussion

The research findings reflect the dialectical nature of the relationship between theory and practice in the context of green accounting adoption and its impact on the quality of sustainability reporting. While theoretical assumptions suggest that green accounting practices represent a positive path to increased environmental transparency, the results reveal a negative direct impact of these practices. This prompts reflection on the gap between companies stated values and their actual implementation. This contradiction may suggest that, in the absence of sound environmental awareness among managers and officials, adopting green accounting may result in a mere formal and ineffective practice. In the same vein, the study concluded that environmental awareness is an important and effective mediating factor, transforming negative impacts into positive drivers for raising the quality of sustainability reporting. This adds a profound philosophical dimension to the second hypothesis. Environmental awareness is more than just knowledge; it is an existential state that motivates organizations to act responsibly, moving beyond formal compliance to a true, substantive commitment to the environment and society. Therefore, engaging in the human and cognitive aspects of leaders is essential and crucial to achieving a positive impact in any green accounting practice. The introduction of legal regulations and technological developments sheds light on how the environment influences organizational behavior, leading to true sustainability. While laws enforce compliance, innovation provides opportunities for environmental conservation. Together, they form a framework that supports environmentally friendly companies and expands understanding. These findings encourage us to view sustainability as a single policy, rather than merely a set of principles, concepts, and infrastructure. In this process, individuals, legislation, and development intertwine within a coordinated framework designed to balance environmental, social, and economic impacts. Some institutions have adopted consistent approaches, while others vary in scope and complexity. However, the overarching goal of sustainable prosperity unites all actors, coordinated by supporting institutions.

## 6. Conclusion

This study’s findings is a concrete indication of the complex dynamics between the adoption of green accounting practices and the quality of sustainability reporting. The study found a significant negative correlation between implementation intensity and reporting standards, suggesting a gap between targets and actual implementation. In contrast, the study found that senior management’s environmental awareness played a key mediating role, transforming this negative effect into a significant positive impact on reporting. This confirms that environmental awareness requires more than just knowledge; it is fundamental to the effective implementation of principled sustainability commitments across all corporate functions. The study also highlights the importance of specific environmental factors, with laws, regulations, and technological advances having a significant and profound impact on the model’s explanatory power. This underscores the critical importance of institutional and technological infrastructure for the success of any green accounting initiative. The limited sample size of the Central Bank of Iraq may limit the generalizability of the findings to other industries or regions. The study also faced challenges in collecting accurate data on green accounting practices and environmental awareness due to the confidentiality and sensitivity of certain information. Furthermore, in some cases, unobserved cultural and organizational differences may have influenced the results. Based on these findings, policymakers are advised to strengthen managerial awareness and training on the importance of environmental awareness, establish policy frameworks requiring organizations to implement effective green accounting standards, and invest in modernizing accounting technologies and digital systems to facilitate the measurement and disclosure of environmental data. They should also promote a corporate culture that prioritizes sustainability through incentives and clear environmental performance indicators. Looking ahead, it is recommended to expand the scope of research to include different economic sectors and examine how corporate culture and ethical behavior, as influencing variables, influence the implementation of green accounting. Furthermore, it is recommended to use qualitative methods to deepen the understanding of contextual influences and conduct comparative analysis across different regulatory and legal environments to enhance the generalizability of the findings. Overall, this research highlights the importance of integrating environmental education, legal structures, and technological advancements to achieve meaningful sustainability through high-quality reporting. This requires a holistic approach that considers human and organizational dimensions in addition to technical capabilities.

### Underlying data

Repository Name: [Data and statistical outputs related to Evaluating the Impact of Adopting Green Accounting on Sustainability Reporting at the Central Bank of Iraq (CBI): A Vision to Support the 2030 Sustainable Development Goals (SDGs)]. https://doi.org/10.5281/zenodo.18098391 ^24^

The project contains the following Underlying data:

- [ **Questionnaire List.xlsx**] (This file contains a questionnaire list designed using a five-point Likert scale, including variables, indicators, and items).
- [ **SmartPLS Report with Control Variable.xlsx**] (This file contains the data and outputs of the statistical analysis before the control variables were added to the mathematical model to examine their impact on the model’s explanatory quality).
- [ **SmartPLS Report without Control Variable.xlsx**] (This file contains the data and outputs of the statistical analysis processed in SmartPLS after the control variables were added to the mathematical model).
