# Supplementary material for: Evaluating the Impact of Adopting Green Accounting on Sustainability Reporting at the Central Bank of Iraq (CBI): A Vision to Support the 2030 Sustainable Development Goals (SDGs)
Source: F1000Res. 2026 Jan 27;15:122. [Version 1] doi: 10.12688/f1000research.175287.1 (PMC13069382; doi:10.12688/f1000research.175287.1)
Supplement: Peer review history

# Reviewer report: Reviewer response for version 1

**DOI:** 10.5256/f1000research.193257.r469329

**Published:** 2026-04-10

**Authors:** Nahariah Binti Jaffar

**Affiliations:** 1 Putra Business School, Selangor, Selangor, Malaysia

This study is imperative as it integrated green accounting, environmental awareness and

 sustainability reporting in support the Sustainable Development Goals and Vision 2030. The novelty of the study is shown through its focus on the Central Bank of Iraq which is viewed to have strategic potential to act as a catalyst for green transformation.

**Title**

 The authors may consider to include the mediator (environmental awareness) in the title. By doing so, the research focus would be well reflected in the title.

**Abstract**

 Under the “Purpose” section, the authors stated “It examined the moderating effect of environmental awareness on this relationship”. It supposed to be “mediating” not “moderating”. Please check.

**Introduction**

 This article addressed an important topic of green accounting and sustainability reporting with the environmental awareness as the mediator. The issue of inadequacy of the traditional financial reporting to capture environmental and social dimension of performance is clearly articulated. Green accounting was established to recognise environmental cost which can enhance the quality of sustainability reporting.

 Overall, the introduction section discussed background information that is relevant to the focus of the study. The discussion offered a clear background that led to the research gap. As such, the background information demonstrates the significance and worthiness of the study.

 It is suggested that the article explicitly addressed the research question/s.

**Literature Review**

 The authors should be credited as most of the references are from the current five years literature. However, more references should be added on the key variables namely green accounting, environment awareness and sustainability reporting. Additionally, underpinning theory was not introduced to support the conceptual framework of the study. It is highly recommended for scientific research to be supported by theory/s to explain the relationships among the variables.

**Conceptual framework**

 It is suggested that the study consistently use a particular term, i.e. conceptual framework, and not interchangeably use with theoretical framework and theoretical model. On the conceptual framework diagram, it looks like the path model produced by the SmartPls. It is very common that measurement items not being displayed in a conceptual framework diagram.    

 The inclusion of legal regulations and level of technology in the conceptual framework for a survey type of research contribute to the comprehensiveness of the framework.

**Hypotheses development**

 Certain parts of the third paragraph on the development of hypothesis 2 requires some revision because they addressed the board characteristics effects on environmental awareness rather than the role of environmental awareness itself. Furthermore, the development of hypothesis 2 is lack of literature and theory supports. Therefore, more past studies should be included in the development of hypothesis 2. Also, theory/s application is not included in the development of all hypotheses. It is suggested that the word “influences” in hypothesis 2 be changed with the word “mediates” to clearly depict the role of the environmental awareness as a mediator.

**Research Method**

 The research method is well described. However, on the **Population and sampling:**

 Please revise this statement on unit of analysis: “The data collected from these individuals

 formed the unit of analysis.” This statement is not accurate; it is interpreted as the data is the unit of analysis. While, the unit of analysis of the study is the individual personnel.

 It is suggested that for Table 2: Name the first column as type of variable and column 2 as Variable name and measurement items.   

**Data Analysis**

 Appropriate tools and analysis have been performed on the study data using the Smartpls software.  

**Discussion**

 The discussion of findings offers important insights about the effect of green accounting on sustainability reporting and the mediating role of environmental awareness on this relationship. It is imperative if comparison of the study’s findings with previous studies is made and provide justification. Furthermore, when underpinning is introduced, the discussion should also include the alignment of the findings with the theory and provide justification.

Is the work clearly and accurately presented and does it cite the current literature?

Yes

If applicable, is the statistical analysis and its interpretation appropriate?

Yes

Are all the source data underlying the results available to ensure full reproducibility?

Yes

Is the study design appropriate and is the work technically sound?

Yes

Are the conclusions drawn adequately supported by the results?

Yes

Are sufficient details of methods and analysis provided to allow replication by others?

Yes

Reviewer Expertise:

Financial accounting and reporting, auditing

I confirm that I have read this submission and believe that I have an appropriate level of expertise to confirm that it is of an acceptable scientific standard.

# Reviewer report: Reviewer response for version 1

**DOI:** 10.5256/f1000research.193257.r464825

**Published:** 2026-03-19

**Authors:** Prof. Dr. Iman Shakir Mohammed

**Affiliations:** 1 Accounting Department, College of Administration and Economic , Al-Iraqia University, Baghdad, Iraq, Baghdad, Iraq

Evaluating the Impact of Adopting Green Accounting on Sustainability Reporting at the Central Bank of Iraq (CBI): A Vision to Support the 2030 Sustainable Development Goals (SDGs)

1. This study is distinguished by its originality, methodological rigor, and robust practical results, making a significant contribution to the literature by revealing the discrepancy between the symbolic adoption of green accounting and its actual practical outcomes. It also highlights the crucial role of environmental awareness, technology, and legislation in enhancing the effectiveness of this approach. The direct link between green accounting and the quality of sustainable reporting within the framework of the 2030 Sustainable Development Goals is a genuine strategic vision that reflects profound thinking and a sophisticated understanding of the current stage's requirements.
2. The abstract is strong and coherent, covering all essential elements, including the objective, methodology, results, practical applications, and keywords. It makes a valuable contribution to the literature by revealing the direct negative impact of green accounting in the absence of environmental awareness, while highlighting the crucial mediating role of leadership awareness in enhancing effectiveness. However, the abstract does not address the limitations or challenges, which is an important element for strengthening the research's credibility.
3. **The research aimed** to clearly define the relationship between green accounting and the quality of sustainable reporting, while introducing environmental awareness as a mediating factor that contributes to explaining and strengthening this relationship. Furthermore, the direct link to Vision 2030 and the Sustainable Development Goals reflects the originality of this approach and its importance to international policies, thus enhancing the scientific and practical value of the study.
4. **The introduction** is strong and coherent, placing the research within a clear global and local context.

• Originality is evident through its focus on the role of the Central Bank of Iraq, an area that has not been studied in depth.

 • It simply needs to strengthen its references and provide a clearer link to the SDGs.

**5.The literature review** is strong in terms of organization and theoretical coherence, and it clearly highlights the research gap.• It needs to be strengthened with more references, critical analysis, and a clearer connection to the SDGs and the Iraqi context to become more in-depth and original.

- Although the Central Bank of Iraq is mentioned, the methodology does not clarify how it addresses the specificities of the local context (such as weak technological infrastructure or the legal framework).
- **6.  Sample and Procedure Details:**

• Sampling Methodology: The sampling method (purposive, stratified, random) was not specified, nor was the final response rate.

 • Critical Analysis of Demographic Characteristics: It would be better to clarify the participants' characteristics (experience, job level) to strengthen the validity of the results.

 • Statistical Analysis: The software used (e.g., AMOS or Smart PLS) was not specified. The hypotheses are written in general terms and need to be formulated more precisely. It would be better to formulate them in a statistically testable way (e.g., "There is a statistically significant positive relationship between the adoption of green accounting and the quality of sustainable reporting").

**7. Results and Discussion**• The analysis highlights the discrepancy between theoretical expectations, which assume a positive impact of green accounting, and practical results, which reveal a direct negative impact. This reflects scientific originality and adds new value to the literature.

 • Introducing the philosophical dimension of environmental awareness as a mediating factor lends the study academic depth and underscores that knowledge alone is insufficient; it must be transformed into an existential and behavioral commitment.

 • Linking laws and technology as supporting factors strengthens the interpretation and provides the model with a comprehensive practical framework.

 • The results reveal the direct negative impact of adopting green accounting on the quality of sustainable reporting in the absence of environmental awareness. This is an original conclusion that adds value to the literature.

 • The crucial mediating role of environmental awareness and leadership in transforming negative impacts into positive ones is highlighted, reinforcing the importance of conscious leadership in organizations.

 • The results demonstrate that the legal and technological infrastructure significantly enhances the explanatory power of the model and supports its practical applicability.

 • The study offers practical recommendations for policymakers in Iraq, including: promoting environmental awareness, developing legal frameworks, investing in technology, and building a sustainable corporate culture. The results are directly linked to Iraq's Vision 2030 and the Sustainable Development Goals, giving them strategic importance at the national and international policy levels.

Is the work clearly and accurately presented and does it cite the current literature?

Yes

If applicable, is the statistical analysis and its interpretation appropriate?

Yes

Are all the source data underlying the results available to ensure full reproducibility?

Yes

Is the study design appropriate and is the work technically sound?

Yes

Are the conclusions drawn adequately supported by the results?

Yes

Are sufficient details of methods and analysis provided to allow replication by others?

Yes

Reviewer Expertise:

Financial accounting and auditing, and government accounting

I confirm that I have read this submission and believe that I have an appropriate level of expertise to confirm that it is of an acceptable scientific standard.
